# Butallyl­onal 1,4-dioxane hemisolvate

**DOI:** 10.1107/S1600536810038651

**Published:** 2010-09-30

**Authors:** Thomas Gelbrich, Denise Rossi, Ulrich J. Griesser

**Affiliations:** a Institute of Pharmacy, University of Innsbruck, Innrain 52, 6020 Innsbruck, Austria

## Abstract

The asymmetric unit of the title compound [systematic name: 5-(1-bromo­prop-2-en-1-yl)-5-*sec*-butyl­pyrimidine-2,4,6-trione 1,4-dioxane hemisolvate], C_11_H_15_BrN_2_O_3_·0.5C_4_H_8_O_2_, contains one half-mol­ecule of 1,4-dioxane and one mol­ecule of butallyl­onal, with an almost planar barbiturate ring [largest deviation from the mean plane = 0.049 (5) Å]. The centrosymmetric dioxane mol­ecule adopts a nearly ideal chair conformation. The barbiturate mol­ecules are linked together by an N—H⋯O hydrogen bond, giving a single-stranded chain. Additionally, each dioxane mol­ecule acts as a bridge between two anti­parallel strands of hydrogen-bonded barbiturate mol­ecules *via* two hydrogen bonds, N—H⋯O(dioxane)O⋯H—N. Thus, a ladder structure is obtained, with the connected barbiturate mol­ecules forming the ‘stiles’ and the bridging dioxane mol­ecules the ‘rungs’.

## Related literature

For the preparation of butallyl­onal, see: J. D. Riedel Akt.-Ges. (1924 ▶); Boedecker (1929 ▶). For related structures, see: Al-Saqqar *et al.* (2004 ▶); Gelbrich *et al.* (2007 ▶, 2010 ▶); Craven *et al.* (1969 ▶); Gatehouse & Craven (1971 ▶); Lewis *et al.* (2004 ▶); Zencirci *et al.* (2009 ▶). For hydrogen-bond motifs, see: Bernstein *et al.* (1995 ▶).
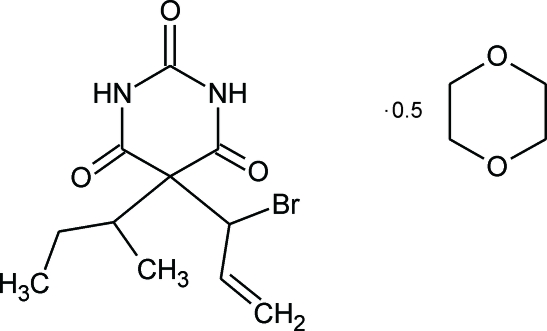


## Experimental

### 

#### Crystal data

C_11_H_15_BrN_2_O_3_·0.5C_4_H_8_O_2_
*M* *_r_* = 347.21
Monoclinic, 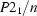

*a* = 10.494 (2) Å
*b* = 6.7679 (8) Å
*c* = 21.864 (3) Å
β = 97.294 (15)°
*V* = 1540.3 (4) Å^3^
*Z* = 4
Mo *K*α radiation
μ = 2.68 mm^−1^
*T* = 293 K
0.25 × 0.08 × 0.07 mm

#### Data collection

Oxford Diffraction Xcalibur Ruby Gemini ultra diffractometer
Absorption correction: multi-scan (*CrysAlis PRO*; Oxford Diffraction, 2007 ▶) *T*_min_ = 0.990, *T*_max_ = 1.000
9189 measured reflections
2714 independent reflections
1171 reflections with *I* > 2σ(*I*)
*R*_int_ = 0.100

#### Refinement

*R*[*F* ^2^ > 2σ(*F* ^2^)] = 0.064
*wR*(*F* ^2^) = 0.145
*S* = 0.95
2714 reflections
189 parameters
2 restraints
H atoms treated by a mixture of independent and constrained refinement
Δρ_max_ = 0.56 e Å^−3^
Δρ_min_ = −0.33 e Å^−3^

### 

Data collection: *CrysAlis PRO* (Oxford Diffraction, 2007 ▶); cell refinement: *CrysAlis PRO*; data reduction: *CrysAlis PRO*; program(s) used to solve structure: *SHELXS97* (Sheldrick, 2008 ▶); program(s) used to refine structure: *SHELXL97* (Sheldrick, 2008 ▶); molecular graphics: *XP* in *SHELXTL* (Sheldrick, 2008 ▶) and *Mercury* (Bruno *et al.*, 2002 ▶); software used to prepare material for publication: *publCIF* (Westrip, 2010 ▶).

## Supplementary Material

Crystal structure: contains datablocks I, global. DOI: 10.1107/S1600536810038651/fj2340sup1.cif

Structure factors: contains datablocks I. DOI: 10.1107/S1600536810038651/fj2340Isup2.hkl

Additional supplementary materials:  crystallographic information; 3D view; checkCIF report

## Figures and Tables

**Table 1:** Hydrogen-bond geometry (Å, °)

*D*—H⋯*A*	*D*—H	H⋯*A*	*D*⋯*A*	*D*—H⋯*A*
N1—H1⋯O4^i^	0.88 (1)	1.98 (2)	2.837 (5)	166 (6)
N3—H3⋯O1*S*	0.89 (4)	1.87 (4)	2.757 (6)	177 (5)

Symmetry code: (i) 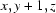.

**Fig. 1.**
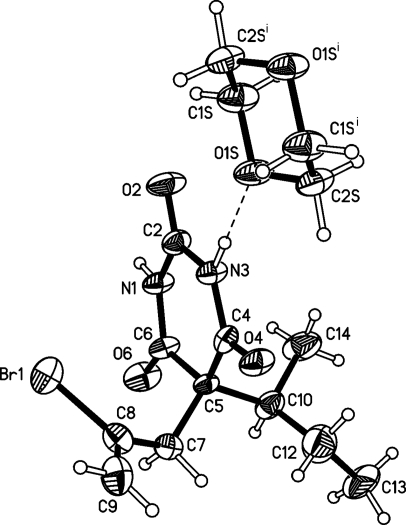
The molecular structures of (I) with displacement ellipsoids drawn at the 30% probability level. Hydrogen atoms are shown as spheres of arbitrary size. Symmetry code: (i) -x, -y, -z.

**Fig. 2.**
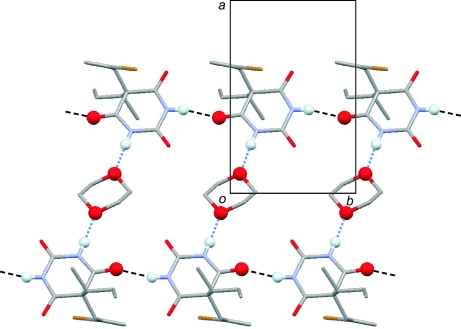
A portion of the one-dimensional hydrogen bonded ladder structure which consists of two antiparallel strands of singly N–H···O bonded barbiturate molecules, which are bridged by dioxane molecules, N–H···O(dioxane)O···H–N. The structure is viewed parallel to the c-axis. O and H atoms involved in hydrogen bonding ar drawn as balls.

## supplementary crystallographic information

### Comment

5,5-Dihydroxybarbituric acid (alternative names: butallylonal, butylalylonal, pernocton, pernoston, sonbutal; CAS number 1142–70-7) has been used as a sedative drug since the 1920s, mainly as an anaesthetic in veterinary medicine. The asymmetric unit of the title compound contains one butallylonal molecule exhibiting an almost planar barbiturate ring [where atom C6 shows the largest deviation from the mean plane, 0.049 (5) Å] and half a molecule of 1,4-dioxane. The two torsion angles of the C8—C7—C5—C10—C14 chain are *trans*, C7—C5—C10—C12 is *gauche* and C5—C10—C14 *trans* (see Fig. 1). The centrosymmetric dioxane molecule adopts a near-to-ideal chair conformation.

The barbiturate molecules are linked together by one N—H···O bond to give a single-stranded chain. Additionally, each dioxane molecule acts as a bridge between two antiparallel strands of H-bonded barbiturate molecules. This interaction involves two hydrogen bonds, N—H···O(dioxane)O···H—N. Overall, a ladder structure is generated, which propagates parallel to the *b* axis (see Fig. 2). The stiles of the ladder are formed by the connected barbiturate molecules and its rungs by the bridging dioxane molecules. This H-bonded structure is reminiscent of the ladder motif observed in single component structures of several barbiturates, see Craven *et al.* (1969); Gatehouse & Craven (1971); Lewis *et al.* (2004); Gelbrich *et al.* (2007); Zencirci *et al.* (2009). The main difference to the title structure is that in these cases, a second C=O group participates in hydrogen bonding so that two antiparallel strands of H-bonded barbiturate molecules are linked together directly *via* centrosymmetric *R*_2_^2^(8) rings (Bernstein *et al.*, 1995).

### Experimental

A solution of butallylonal ("Pernocton"; J. D. Riedel - E. de Haën AG, Berlin) in 1,4-dioxane was filled into an NMR tube and left for evaporation. Colourless crystals of the title compound were obtained after several weeks.

### Refinement

All H atoms were identified in a difference map. H atoms bonded to C atoms were positioned geometrically and refined with *U*_iso_(H) = 1.2 *U*_eq_(C). Hydrogen atoms attached to N were refined with restrained distances [N—H = 0.88 (2) Å], and their *U*_iso_ parameters were refined freely.

### Crystal data

**Table:**

C_11_H_15_BrN_2_O_3_·0.5C_4_H_8_O_2_	*F*(000) = 712
*M**_r_* = 347.21	*D*_x_ = 1.497 Mg m^−^^3^
Monoclinic, *P*2_1_/*n*	Mo *K*α radiation, λ = 0.71073 Å
Hall symbol: -P 2yn	Cell parameters from 837 reflections
*a* = 10.494 (2) Å	θ = 2.5–28.5°
*b* = 6.7679 (8) Å	µ = 2.68 mm^−^^1^
*c* = 21.864 (3) Å	*T* = 293 K
β = 97.294 (15)°	Prism, colourless
*V* = 1540.3 (4) Å^3^	0.25 × 0.08 × 0.07 mm
*Z* = 4	

### Data collection

**Table:**

Oxford Diffraction Xcalibur Ruby Gemini ultra diffractometer	2714 independent reflections
Radiation source: Enhance Ultra (Cu) X-ray Source	1171 reflections with *I* > 2σ(*I*)
mirror	*R*_int_ = 0.100
Detector resolution: 10.3575 pixels mm^-1^	θ_max_ = 25.1°, θ_min_ = 3.2°
ω scans	*h* = −10→12
Absorption correction: multi-scan (*CrysAlis PRO*; Oxford Diffraction, 2007)	*k* = −8→8
*T*_min_ = 0.990, *T*_max_ = 1.000	*l* = −25→26
9189 measured reflections	

### Refinement

**Table:**

Refinement on *F*^2^	Primary atom site location: structure-invariant direct methods
Least-squares matrix: full	Secondary atom site location: difference Fourier map
*R*[*F*^2^ > 2σ(*F*^2^)] = 0.064	Hydrogen site location: inferred from neighbouring sites
*wR*(*F*^2^) = 0.145	H atoms treated by a mixture of independent and constrained refinement
*S* = 0.95	*w* = 1/[σ^2^(*F*_o_^2^) + (0.0409*P*)^2^] where *P* = (*F*_o_^2^ + 2*F*_c_^2^)/3
2714 reflections	(Δ/σ)_max_ < 0.001
189 parameters	Δρ_max_ = 0.56 e Å^−^^3^
2 restraints	Δρ_min_ = −0.33 e Å^−^^3^

### Special details

**Table:**

Experimental. CrysAlisPro, Oxford Diffraction Ltd., Empirical absorption correction using spherical harmonics, implemented in SCALE3 ABSPACK scaling algorithm.
Geometry. All e.s.d.'s (except the e.s.d. in the dihedral angle between two l.s. planes) are estimated using the full covariance matrix. The cell e.s.d.'s are taken into account individually in the estimation of e.s.d.'s in distances, angles and torsion angles; correlations between e.s.d.'s in cell parameters are only used when they are defined by crystal symmetry. An approximate (isotropic) treatment of cell e.s.d.'s is used for estimating e.s.d.'s involving l.s. planes.
Refinement. Refinement of *F*^2^ against ALL reflections. The weighted *R*-factor *wR* and goodness of fit *S* are based on *F*^2^, conventional *R*-factors *R* are based on *F*, with *F* set to zero for negative *F*^2^. The threshold expression of *F*^2^ > σ(*F*^2^) is used only for calculating *R*-factors(gt) *etc*. and is not relevant to the choice of reflections for refinement. *R*-factors based on *F*^2^ are statistically about twice as large as those based on *F*, and *R*- factors based on ALL data will be even larger.

### Fractional atomic coordinates and isotropic or equivalent isotropic displacement parameters (Å^2^)

**Table:**

	*x*	*y*	*z*	*U*_iso_*/*U*_eq_	
Br1	0.64263 (9)	0.24500 (13)	0.04609 (4)	0.0951 (4)	
N1	0.4336 (5)	0.4954 (6)	0.1405 (2)	0.0504 (15)	
H1	0.435 (6)	0.6246 (17)	0.138 (2)	0.061*	
N3	0.3225 (5)	0.2096 (6)	0.1096 (2)	0.0468 (14)	
H3	0.253 (3)	0.163 (7)	0.087 (2)	0.056*	
O2	0.2482 (5)	0.5076 (6)	0.0762 (2)	0.0786 (16)	
O6	0.6264 (5)	0.4897 (5)	0.1981 (2)	0.0752 (16)	
O4	0.3920 (4)	−0.0903 (5)	0.13692 (18)	0.0579 (13)	
C2	0.3306 (7)	0.4133 (8)	0.1065 (3)	0.0519 (18)	
C4	0.4088 (6)	0.0884 (8)	0.1410 (3)	0.0450 (16)	
C5	0.5229 (6)	0.1748 (6)	0.1812 (3)	0.0386 (15)	
C6	0.5334 (7)	0.4010 (8)	0.1731 (3)	0.0505 (18)	
C7	0.6478 (6)	0.0760 (8)	0.1667 (3)	0.0501 (17)	
H7A	0.7191	0.1616	0.1817	0.060*	
H7B	0.6588	−0.0463	0.1899	0.060*	
C8	0.6567 (6)	0.0305 (9)	0.1013 (3)	0.0606 (19)	
C9	0.6782 (7)	−0.1521 (11)	0.0779 (3)	0.084 (2)	
H9A	0.6886	−0.2609	0.1040	0.101*	
H9B	0.6823	−0.1675	0.0360	0.101*	
C10	0.5050 (7)	0.1407 (8)	0.2512 (3)	0.0605 (19)	
H10	0.5815	0.1959	0.2756	0.073*	
C12	0.4966 (9)	−0.0656 (10)	0.2707 (4)	0.097 (3)	
H12A	0.5544	−0.1461	0.2499	0.117*	
H12B	0.4099	−0.1140	0.2593	0.117*	
C13	0.5331 (10)	−0.0833 (13)	0.3418 (3)	0.129 (4)	
H13D	0.6067	−0.0020	0.3546	0.193*	
H13E	0.5530	−0.2184	0.3525	0.193*	
H13F	0.4622	−0.0404	0.3622	0.193*	
C14	0.3888 (8)	0.2595 (10)	0.2694 (3)	0.103 (3)	
H14A	0.4032	0.3982	0.2640	0.154*	
H14B	0.3793	0.2338	0.3117	0.154*	
H14C	0.3120	0.2201	0.2436	0.154*	
O1S	0.1014 (4)	0.0764 (5)	0.04030 (19)	0.0650 (14)	
C1S	0.0085 (7)	0.2010 (7)	0.0056 (3)	0.072 (2)	
H1S1	0.0491	0.3243	−0.0036	0.086*	
H1S2	−0.0595	0.2313	0.0303	0.086*	
C2S	0.0465 (7)	−0.1081 (8)	0.0515 (3)	0.066 (2)	
H2S1	−0.0200	−0.0899	0.0781	0.080*	
H2S2	0.1119	−0.1938	0.0727	0.080*	

### Atomic displacement parameters (Å^2^)

**Table:**

	*U*^11^	*U*^22^	*U*^33^	*U*^12^	*U*^13^	*U*^23^
Br1	0.1010 (8)	0.1162 (6)	0.0699 (6)	0.0078 (6)	0.0173 (5)	0.0284 (5)
N1	0.050 (4)	0.025 (2)	0.070 (3)	−0.006 (3)	−0.019 (3)	0.003 (3)
N3	0.048 (4)	0.025 (2)	0.062 (3)	−0.006 (2)	−0.014 (3)	0.002 (2)
O2	0.068 (4)	0.046 (2)	0.112 (4)	0.003 (2)	−0.028 (3)	0.021 (2)
O6	0.072 (4)	0.051 (2)	0.092 (4)	−0.017 (2)	−0.032 (3)	−0.008 (2)
O4	0.063 (3)	0.027 (2)	0.078 (3)	−0.0022 (19)	−0.011 (2)	0.0016 (19)
C2	0.056 (5)	0.037 (3)	0.061 (4)	0.004 (3)	0.000 (4)	0.009 (3)
C4	0.057 (5)	0.036 (3)	0.042 (4)	−0.002 (3)	0.006 (3)	0.006 (3)
C5	0.043 (4)	0.027 (3)	0.044 (4)	0.001 (3)	−0.003 (3)	0.005 (2)
C6	0.059 (5)	0.042 (3)	0.046 (4)	0.009 (4)	−0.011 (4)	0.002 (3)
C7	0.044 (5)	0.047 (3)	0.059 (4)	0.000 (3)	0.003 (3)	−0.001 (3)
C8	0.049 (5)	0.066 (4)	0.068 (5)	−0.003 (4)	0.009 (4)	−0.002 (4)
C9	0.091 (7)	0.098 (5)	0.068 (5)	0.003 (5)	0.031 (5)	0.005 (4)
C10	0.082 (6)	0.050 (3)	0.052 (4)	0.020 (4)	0.017 (4)	0.006 (3)
C12	0.107 (8)	0.084 (5)	0.103 (7)	−0.001 (5)	0.023 (6)	0.008 (5)
C13	0.144 (9)	0.186 (8)	0.060 (6)	0.060 (8)	0.029 (6)	0.072 (6)
C14	0.132 (8)	0.114 (6)	0.067 (5)	0.072 (6)	0.032 (5)	0.019 (5)
O1S	0.058 (3)	0.046 (2)	0.080 (3)	−0.003 (2)	−0.031 (3)	0.001 (2)
C1S	0.071 (5)	0.041 (3)	0.097 (6)	0.006 (3)	−0.016 (4)	−0.002 (4)
C2S	0.073 (6)	0.056 (4)	0.063 (5)	0.004 (4)	−0.014 (4)	0.012 (3)

### Geometric parameters (Å, °)

**Table:**

Br1—C8	1.881 (6)	C10—C12	1.466 (8)
N1—C6	1.351 (7)	C10—C14	1.553 (9)
N1—C2	1.351 (7)	C10—H10	0.9800
N1—H1	0.876 (10)	C12—C13	1.558 (9)
N3—C4	1.344 (6)	C12—H12A	0.9700
N3—C2	1.384 (7)	C12—H12B	0.9700
N3—H3	0.89 (4)	C13—H13D	0.9600
O2—C2	1.203 (6)	C13—H13E	0.9600
O6—C6	1.215 (6)	C13—H13F	0.9600
O4—C4	1.224 (5)	C14—H14A	0.9600
C4—C5	1.510 (7)	C14—H14B	0.9600
C5—C7	1.540 (8)	C14—H14C	0.9600
C5—C6	1.547 (7)	O1S—C2S	1.410 (7)
C5—C10	1.583 (8)	O1S—C1S	1.431 (7)
C7—C8	1.479 (8)	C1S—C2S^i^	1.452 (8)
C7—H7A	0.9700	C1S—H1S1	0.9700
C7—H7B	0.9700	C1S—H1S2	0.9700
C8—C9	1.366 (8)	C2S—C1S^i^	1.452 (8)
C9—H9A	0.9300	C2S—H2S1	0.9700
C9—H9B	0.9300	C2S—H2S2	0.9700
			
C6—N1—C2	127.5 (5)	C14—C10—C5	111.4 (5)
C6—N1—H1	119 (4)	C12—C10—H10	106.3
C2—N1—H1	113 (4)	C14—C10—H10	106.3
C4—N3—C2	126.3 (5)	C5—C10—H10	106.3
C4—N3—H3	121 (3)	C10—C12—C13	110.3 (6)
C2—N3—H3	112 (3)	C10—C12—H12A	109.6
O2—C2—N1	123.6 (5)	C13—C12—H12A	109.6
O2—C2—N3	120.8 (6)	C10—C12—H12B	109.6
N1—C2—N3	115.6 (5)	C13—C12—H12B	109.6
O4—C4—N3	118.9 (5)	H12A—C12—H12B	108.1
O4—C4—C5	121.4 (5)	C12—C13—H13D	109.5
N3—C4—C5	119.6 (4)	C12—C13—H13E	109.5
C4—C5—C7	110.2 (4)	H13D—C13—H13E	109.5
C4—C5—C6	112.3 (5)	C12—C13—H13F	109.5
C7—C5—C6	109.4 (5)	H13D—C13—H13F	109.5
C4—C5—C10	108.8 (5)	H13E—C13—H13F	109.5
C7—C5—C10	110.2 (5)	C10—C14—H14A	109.5
C6—C5—C10	105.9 (4)	C10—C14—H14B	109.5
O6—C6—N1	121.9 (5)	H14A—C14—H14B	109.5
O6—C6—C5	120.2 (5)	C10—C14—H14C	109.5
N1—C6—C5	117.8 (5)	H14A—C14—H14C	109.5
C8—C7—C5	116.7 (5)	H14B—C14—H14C	109.5
C8—C7—H7A	108.1	C2S—O1S—C1S	110.4 (4)
C5—C7—H7A	108.1	O1S—C1S—C2S^i^	111.7 (5)
C8—C7—H7B	108.1	O1S—C1S—H1S1	109.3
C5—C7—H7B	108.1	C2S^i^—C1S—H1S1	109.3
H7A—C7—H7B	107.3	O1S—C1S—H1S2	109.3
C9—C8—C7	125.7 (6)	C2S^i^—C1S—H1S2	109.3
C9—C8—Br1	117.5 (6)	H1S1—C1S—H1S2	107.9
C7—C8—Br1	116.8 (4)	O1S—C2S—C1S^i^	111.1 (5)
C8—C9—H9A	120.0	O1S—C2S—H2S1	109.4
C8—C9—H9B	120.0	C1S^i^—C2S—H2S1	109.4
H9A—C9—H9B	120.0	O1S—C2S—H2S2	109.4
C12—C10—C14	110.0 (7)	C1S^i^—C2S—H2S2	109.4
C12—C10—C5	116.0 (5)	H2S1—C2S—H2S2	108.0

Symmetry codes: (i) −*x*, −*y*, −*z*.

### Hydrogen-bond geometry (Å, °)

**Table:**

*D*—H···*A*	*D*—H	H···*A*	*D*···*A*	*D*—H···*A*
N1—H1···O4^ii^	0.88 (1)	1.98 (2)	2.837 (5)	166 (6)
N3—H3···O1S	0.89 (4)	1.87 (4)	2.757 (6)	177 (5)

Symmetry codes: (ii) *x*, *y*+1, *z*.
